# Analysis of Closed Claims in Cardiovascular Medicine: Importance of Nonclinical Factors

**DOI:** 10.1016/j.jacadv.2025.102467

**Published:** 2025-12-22

**Authors:** Fred Kusumoto, David M. Dudzinski, Jacqueline Ross, Sunny Jhamanni, Daniel K. Cassavar, Richard A. Chazal

**Affiliations:** a Heart Rhythm Division, Department of Cardiovascular Disease, Mayo Clinic, Jacksonville Florida, USA; b Harvard Medical School, Department of Cardiology, Massachusetts General Hospital, Boston, Massachusetts, USA; c The Doctors Company, Napa, California, USA; d Tri-City Cardiology, Chandler, Arizona, USA; e Lee Health Heart Institute, Fort Myers, Florida, USA

**Keywords:** closed claims, communication, medical professional liability

## Abstract

**Background:**

Medical professional liability claims are a marker of conflict between a patient and a health care provider.

**Objectives:**

The purpose of this study was to evaluate the contributing factors (CFs) associated with medical professional liability claims.

**Methods:**

Seven hundred sixty-four cardiovascular-related closed claims with 1,945 CFs were identified from a large insurer (The Doctor’s Company) from 2010 to 2023.

**Results:**

Three clinical CFs: technical performance (329; 43% of claims), patient assessment (242; 32%), and management/selection of therapy (216; 28%) were the most frequently cited CFs. Patient factors, mainly due to nonadherence to medications or instructions, were identified in 171 claims (22%). Nonclinical CFs (987) were more frequent than clinical CFs (787) and patient factors (patient: 171). Nonclinical CFs were very diverse but the most frequent were communication between providers and patient (20%), communication among providers (16%), insufficient documentation (11%), and off-shift/weekend hours (10%). Payment was less common in claims with technical performance or patient factor CFs and more common in claims with patient assessment, selection/management of therapy, and in the 4 most frequent nonclinical CFs. Nonclinical CFs were more commonly observed in claims with payment (1.1/claim) when compared to claims without (0.64/claim). No differences among the different cardiovascular subspecialties were identified. Closed claims were identified in 3% of covered cardiovascular providers over the last reliable portion of the study period.

**Conclusions:**

Clinical and nonclinical CFs are equally important for malpractice claims. While focusing on clinical quality is important, implementing strategies that also account for nonclinical issues, with a particular focus on communication, documentation, and off-shift/weekend coverage could have significant benefits.

Significant patient dissatisfaction with medical care can ultimately lead to medical professional liability (MPL) claims. Analysis of deeply coded MPL claims can provide insight into patient concerns on processes, safety, and quality, and potentially identify strategies to improve relationships between the patients, health care systems, and health care practitioners.^1^ Cardiovascular-related (diagnostic cardiology, interventional cardiology, and cardiac surgery) MPL claims were evaluated to identify contemporary trends and characteristics. Specifically, the analysis was focused on the contributing factors (CFs) that led to the question of negligence or major injury involved in the claim.

## Methods

A retrospective, descriptive study of The Doctor’s Company (TDC) cardiovascular-related closed claims of loss years 2010-2024 was done. Claims with primary responsible services of diagnostic cardiology, interventional cardiology, and cardiac surgery were included. TDC is a large independent physician-owned MPL insurer that provides coverage to physicians in multiple specialties across all 50 states and the District of Columbia. Included in the claims data are claims from Healthcare Risk Advisors which is a subsidiary of TDC that provides MPL for large institutions and academic centers mainly located in the Northeastern United States. An MPL “claim” was defined as an allegation of liability by a patient against an insured physician with a requested for compensation. A “closed claim” was defined as a claim for which no further plaintiff action was possible, including dropped or withdrawn, denied, dismissed, litigated (eg, with jury verdict), or settled. The study was reviewed by the Mayo Clinic Institutional Review Board and in accordance with the Code of Federal Regulations, 45 CFR 46.102. It was determined, based on the nature of the study with the use of deidentified, aggregated data, that Institutional Review Board approval was not required.

An evidence-based clinical coding taxonomy (Controlled Risk Insurance Company, Clinical Coding Taxonomy, V4.0, 2021) was used to code every closed claim.^2^ The taxonomy includes 22 areas for data input (major allegation, responsible service, admitting service, roles, initial diagnosis, final diagnosis, medications, site, location, severity, comorbidities, claimant type, presenting procedure, involved procedure, major injuries, disclosure, apology, CFs, serious reportable events, primary drivers, and clinical description). Each closed claim was analyzed by registered nurse patient safety analysts through review of various MPL case records, including defense and plaintiff experts’ testimony, medical records, plaintiff and defendant depositions, and health care provider interviews. Each closed claim features a clinical summary that provides a succinct narrative of the event and validation for the primary driver and each CF chosen. Cases often have multiple injuries, responsible services, and CFs. CF subcategories were classified as clinical (health care provider with direct responsibility such as technical performance of a procedure or surgery, patient assessment, or selection/management of therapy), patient factors, or nonclinical (not related to direct clinical care by the health care provider). The quality of the TDC coded data has been substantiated through scheduled internal and external audits.

Two-tailed z-test for proportions and Fisher exact probability test were used for comparing groups and the Mantel-Haenszel chi-square test when testing for trends. A *P* < 0.01 was considered significant. All analyses were performed with SAS statistical software, version 9.4 (SAS Institute, Inc).

## Results

Over the study period, 764 cardiovascular-related claims were evaluated (450 classified as high severity, 249 as medium severity, and 65 as low severity) with progressive decrease over the study period (Supplemental Figure 1). Through the study period, the composition of covered cardiovascular providers remained stable (60% invasive cardiologists, 30% cardiologists, and 10% cardiothoracic surgeons). The mean age of the patient involved was 61 ± 13 years (range from birth to 98 years old) with 58% male (Table 1). A total of 183 claims (24%) were from the outpatient setting (162 patient office, 13 ambulatory surgery centers, 8 other) and the remainder were in the hospital: 196 cardiac catheterization laboratory, 159 hospital room, 107 operating room or recovery room, 56 intensive care units, 53 in imaging or other specialty procedure areas, with the remainder in miscellaneous hospital locations. The 3 major allegations for the claims were medical treatment (349, 49%), followed by surgical treatment (178, 23%) and diagnosis related (128, 17%).

**Table 1:** Closed Claim Cases Summary of Patient and Case Characteristics (N = 764)

Age (mean 61 y ± 13 y)	
<18 y	11 (1%)
18-64 y	401 (52%)
>64 y	308 (40%)
Unknown	44 (6%)
Sex	
Male	445 (58%)
Female	318 (42%)
Unknown	1 (nil)
Injury severity	
High (permanent major or death)	450 (59%)
Medium (permanent minor or temporary major/minor)	249 (33%)
Low (temporary insignificant or emotional only)	65 (9%)
Location	
Special procedure area	239 (31%)
Operating room or recovery room	107 (14%)
Hospital room	160 (21%)
Intensive care unit	56 (7%)
Emergency department	9 (1%)
Radiology/Imaging	10 (1%)
Ambulatory surgery	13 (2%)
Physician office	162 (21%)
Other	8 (1%)
Procedure related	
Yes	436 (57%)
No	328 (43%)
Payment	
Yes	219 (29%)
No	545 (71%)

Values are n (%).

**Figure 1:**
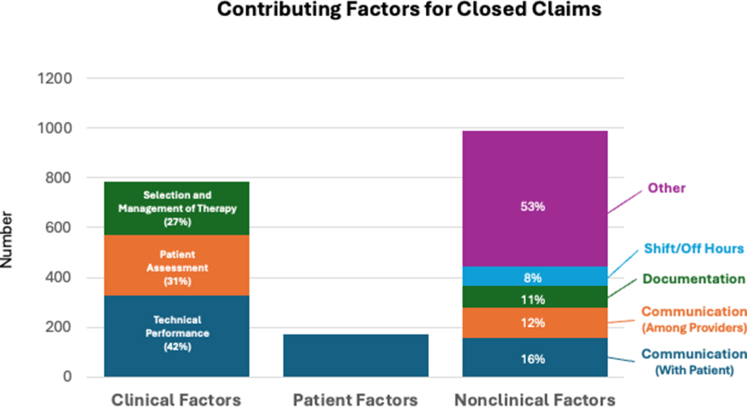
**Contributing Factors Identified During the Study Period Divided Among Clinical Factors, Patient Factors, and Nonclinical Factors** Clinical factors including selection and management of therapy, patient assessment, and technical performance were the largest individual groups. Nonclinical contributing factors were more diverse but collectively were more common than patient factors or clinical factors.

From these 764 claims, 1,945 CFs were identified (claims can have more than one CF) and are summarized in Figure 1, Central Illustration, and Supplemental Figure 2. Clinical CFs were the 3 largest individual groups: technical performance 329 (43%); patient assessment 242 (32%); and selection and management of therapy 216 (28%). Patient factors (171; 22%) mainly due to nonadherence (77% of the patient factor CFs). Nonclinical CFs were a diverse group with the largest contributors being communication issues either between the provider and the patient/family (156; 20%) or communication among providers (123; 16%), documentation issues (86; 11%), and off-hour/weekend conditions (77; 10%). When taken collectively, the 987 nonclinical CFs exceeded clinical CFs (787) and patient factor CF (171). For the most common CF subcategories, specific details are shown in Table 2. When the data were divided into 3 time strata (2010-2014; 2015-2019; 2020-2023), there was no significant change in the overall relative percentages of clinical CFs (34% to 37%), patient factors (8%), and nonclinical CFs (55% to 58%) nor were trends for specific CFs identified (Supplemental Figure 2).

**Central Illustration:**
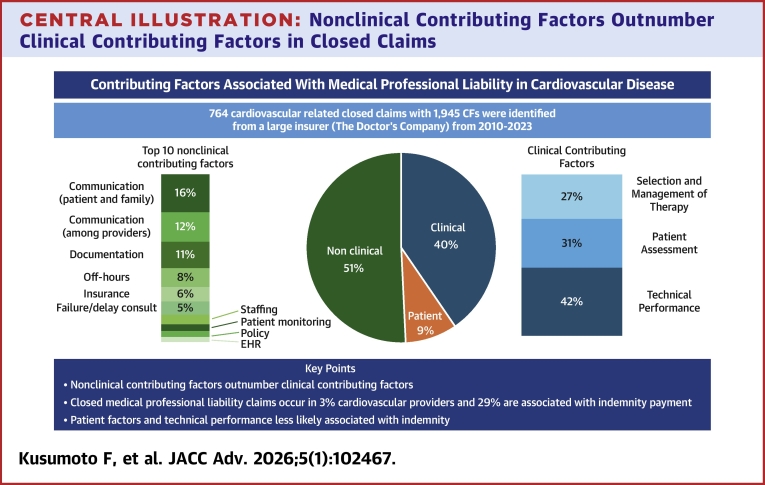
**Nonclinical Contributing Factors Outnumber Clinical Contributing Factors in Closed Claims** The most common nonclinical factors involve communication either with the patient and family or among providers. Focusing on nonclinical factors could reduce medical professional liability claims. CF = contributing factor; EHR = electronic health record.

**Table 2:** Detail of the Most Common Individual Issues Associated With Each Contributing Factor Subcategory

Subcategory	N	Detail (n; %)
Technical performance	329	• Possible technical problem, known complication (n = 307; 93%)
Patient assessment	242	• Failure to appreciate and reconcile relevant sign/symptom/test result (n = 119; 49%) • Failure/delay in ordering diagnostic test (n = 106; 44%)
Selection and management of therapy	216	• Surgical/invasive procedure (n = 118; 55%) • Medical (n = 48; 22%)
Patient factors	171	• Nonadherence with treatment regimen (n = 60; 35%) • Nonadherence with medication (n = 32; n; 19%) • Nonadherence with follow-up call/appointment (n = 31; 18%)
Communication between patient/family and providers	156	• Poor rapport (n = 36; 23%) • Expectations (n = 33; 21%) • Inadequate informed consent for other treatment options (n = 20; 13%)
Communication among providers	123	• Regarding patient’s condition (n = 84; 68%)
Insufficient/lack of documentation	86	• Clinical rationale (n = 22; 26%) • Clinical findings (n = 15; 17%)
Shift/off hours conditions	77	• Weekend/holiday (n = 51; 66%) • Night (n = 26; 34%)

**Figure 2:**
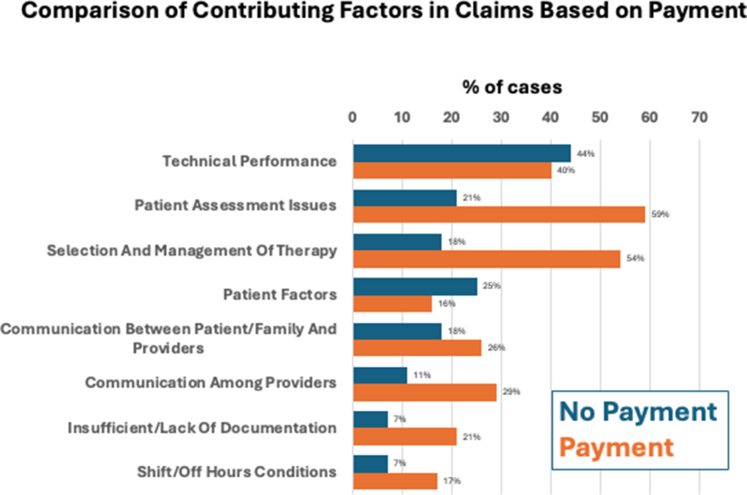
**Different Contributing Factors Based on Whether Associated With Indemnity Payment** Payment more common for patient assessment issues, selection and management of therapy, communication between patient and providers, communication among providers, poor documentation, and shift/off hour conditions. Payment was less common for patient factors.

Two hundred nineteen (29%) of claims resulted in payment. Figure 2 compares common CFs for claims associated with payment vs claims without payment. Claims with CFs due to technical performance or patient factors were less commonly associated with payment when compared to the other CFs. For claims associated with payment, in absolute numbers, the 5 most common (>30) CF-specific details were “possible technical problem with a known complication” (74; 34%), “failure to appreciate and reconcile relevant sign/symptom/test result” (69; 32%), “failure/delay in ordering a diagnostic test” (59; 27%), “communication among providers regarding patient’s condition” (46; 21%), “misinterpretation of diagnostic studies” (35; 16%), and “failure/delay in obtaining consult/referral” (31; 14%). Three hundred forty-nine nonclinical CFs were identified in the 545 claims not associated with payment (0.64 nonclinical CF/claim) compared to 237 nonclinical CFs identified in the claims associated with payment (1.1 nonclinical CF/claim). Claims associated with death were more frequent among those with payment and claims with medium injury were less frequently accompanied with payment (Supplemental Figure 3). No difference between payment or no payment was observed for different clinical or nonclinical locations (Supplemental Table 1).

**Figure 3:**
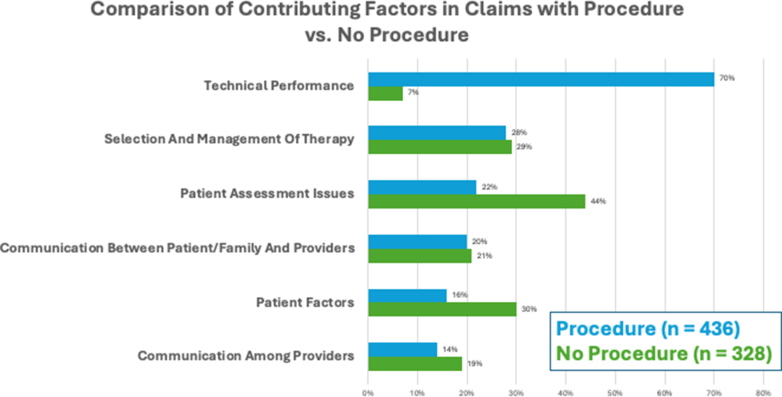
**Most Common Contributing Factors Dependent on Whether Associated With a Procedure or Not** Technical performance more commonly present when a procedure involved, whereas patient assessment and patient factors more common when no procedure involved.

A total of 436 (57%) claims were associated with a procedure or surgery. Claims associated with a procedure or surgery were more likely to have a technical performance CF while patient assessment CFs were most common in those claims not associated with a procedure (Figure 3). A total of 221 claims were associated with a nonsurgical invasive procedure (cardiac catheterization 85, percutaneous coronary intervention 52, and electrophysiology procedure 84). No specific differences in CFs were detected among the 3 procedural types other than selection and management of therapy and patient factors being more commonly observed for percutaneous coronary intervention procedures (Supplemental Figure 4). All nonsurgical procedure types had similar percentages of claims associated with payment (21%-33%) (Supplemental Figure 5).

## Discussion

Our data suggest that the annual risk of a cardiologist facing an MPL claim has decreased over time to approximately 3%. This pattern reflects a general trend that has been observed in other medical specialties.^3^^,^^4^ Specifically, within cardiology in a previous analysis of 781 cardiologists from a large nationwide insurer from 1991 to 2005, the annual risk for a cardiologist to face an MPL claim was 8.6% with 13.6% were associated with an indemnity payment.^5^ In contrast, in our cohort, 29% of cardiovascular-related claims were associated with an indemnity payment. The increased likelihood of a claim being associated with indemnity has been described for all of medicine due to multiple factors including: a strategy to reduce the likelihood of a nuclear verdict (an award >$10 million), increased injury severity, and increased defense costs.^6^, ^7^, ^8^, ^9^

No prior study in cardiology has systematically evaluated CFs associated with closed claims. In the current study, while individual clinical CFs were most frequently observed, when taken collectively nonclinical CFs were more common than clinical CFs. This is a finding that has also been observed when evaluating all medical specialties.^3^

Nonclinical CFs were diverse but the most common were communication between the provider and patient or family, communication among providers, documentation, and off-shift/weekend hours. Although communication has been identified as an important CF associated with MPL claims for over 30 years, the current data show that this aspect of health care continues to be a significant issue.^10^^,^^11^ Documentation also provides an opportunity for improvement and in the current study, poor documentation addressing clinical rationale and clinical findings were the most frequently identified specific issues. A prior study on clinical documentation issues also identified inadequate documentation as a major problem but additionally found inaccurate text, transcription errors, and alterations in documentation.^12^ Consistent use of current electronic health record tools and development of artificial intelligence and other technologies may help make it easier to produce clinical notes that are more accurate and comprehensive and perhaps facilitating more transparent communication among all stakeholders. Finally, implementation of strategies that ensure adequate clinical care during off hours and weekends is another area where medical systems can reduce MPL claims.

Patient factor CFs were observed in 20% of claims and are likely more common in everyday clinical practice. The most common specific issues identified among patient factor CFs were nonadherence to a treatment regimen, medical therapy, or follow-up instructions. Development of postencounter follow-up techniques that “close the loop” and other strategies that have been developed to improve adherence to recommendations would likely help reduce the frequency of this important clinical problem.^13^^,^^14^

The current study found that patient factor CFs in addition to the technical performance clinical CF were less likely to be associated with payment. Clinical CFs such as patient assessment and selection or management of therapy and all nonclinical CFs were more likely to be associated with payment. Finally, claims associated with death were statistically more likely to be associated with payment.

### Study Limitations

There are several limitations to the current study. A closed claim does not necessarily reflect quality of care, though settled/verdict claims may more likely represent deviation from the medical standard of care.^15^ These closed claims data are from only a single insurance provider (although TDC is one of the two largest medical malpractice carriers in the United States). This study cannot compare patient mix, acuity, or volume of individual physicians. It does not account for physician experience, training, or place and setting of employment (however, the focus of this study was evaluating CFs associated with cardiovascular MPL claims rather than assessing clinical outcomes). Although the TDC data include claims from a variety of sources, fewer large medical centers are represented, and some large systems may be self-insured. Finally, closed claims are lagging indicators and closed claims have generally been decreasing thus the largest sample comes from the earlier part of the study period. Nonetheless, analysis of temporal trends did not identify any significant changes in CFs during the study period.

## Conclusions

Clinical issues such as technical performance, patient assessment, and selection and management of therapy remain the most frequent individual CFs for MPL claims involving cardiovascular care. However, a diverse range of nonclinical CFs are commonly observed and collectively are more frequent than clinical factors highlighting the importance of effective systems of care. Nonclinical CFs are more frequently observed in MPL claims associated with indemnity. The most common nonclinical CFs involve communication between patients and health care providers and among health care providers. In addition to improving communication, implementing systems of care that improve documentation and off-hours/weekend clinical care may also reduce MPL claims.

## Funding support and author disclosures

The authors have reported that they have no relationships relevant to the contents of this paper to disclose.
