# Supplementary material for: Analysis of Closed Claims in Cardiovascular Medicine: Importance of Nonclinical Factors
Source: JACC Adv. 2025 Dec 22;5(1):102467. doi: 10.1016/j.jacadv.2025.102467 (PMC12796538; doi:10.1016/j.jacadv.2025.102467)
Supplement: Supplemental_Material [file mmc1.docx]

**American College of Cardiology Medical Professional Liability Task Force:**

**American College of Cardiology**

Richard Chazal MD MACC (Chair)

Cathie Biga MSN MACC (Chair-elect)

Shyam Bhakta, MD, MBA, FACC

Charles L. Brown III MD FACC

Amy Brownell FNP-C FACC

Thomas A. Draper MBA FACC

David Dudzinsky MD JD FACC

John P. Erwin, III, MD, FACC

Edward A Fry MS MACC

Dipti Itchhaporia MD, MACC

Sunny Jhamanni MD FACC

Fred M. Kusumoto, MD, FACC

Jeffrey Kuvin, MD, FACC

Nicole L. Lohr, MD, PhD, FACC

Marc Shelton, MD,FACC

Thad F. Waites, MD, MACC

Brenda Hindle, MS, MBA

**The Doctors Company**

Richard E. Anderson, MD, FACP, Chairman and CEO

Jeff Brady, MD, MPH, Vice President, Patient Safety

Krista Clark, Marketing Project Manager

Debra Davidson, M.J., ARM, CPHRM, CPPS, Senior Patient Safety Risk Manager, Patient Safety & Risk Management

Elizabeth Y. Healy, Vice President, Government and Community Relations

Kendra Heredia, Assistant Vice President, National Business Development

Laura Kline, MBA, CPCU, CIC, Senior Vice President, Business Development and Regional Operating Officer (Region II)

Bryan Lawton, PhD, Chief Governance Officer

Elizabeth Valente, Vice President, Underwriting and Quality Assurance

Todd Virkus, Vice President, Claims

**Supplement Table 1:** Relationship Between Location and Payment vs. No Payment

| **Location** | **No Payment (545)** | **Payment (219)** |
| --- | --- | --- |
| **Hospital** | 420 (77%) | 161 (74%) |
| - Cardiac Catheterization Lab | 151 (36%) | 45 (28%) |
| - Other Procedure | 26 (6%) | 17 (11%) |
| - Surgery (OR, Recovery) | 77 (18%) | 30 (19%) |
| - Intensive Care Unit | 42 (10%) | 14 (9%) |
| - Hospital Room | 111 (26%) | 49 (30%) |
| - Radiology/Imaging | 7 (2%) | 3 (2%) |
| - Emergency | 6 (1%) | 3 (2%) |
| **Outpatient** | 125 (23%) | 58 (26%) |
| - Clinic/Office | 107 (86%) | 55 (95%) |
| - Ambulatory Surgery | 10 (8%) | 3 (5%) |
| - Other | 8 (6%) | 0 |

No significant differences were found among different locations


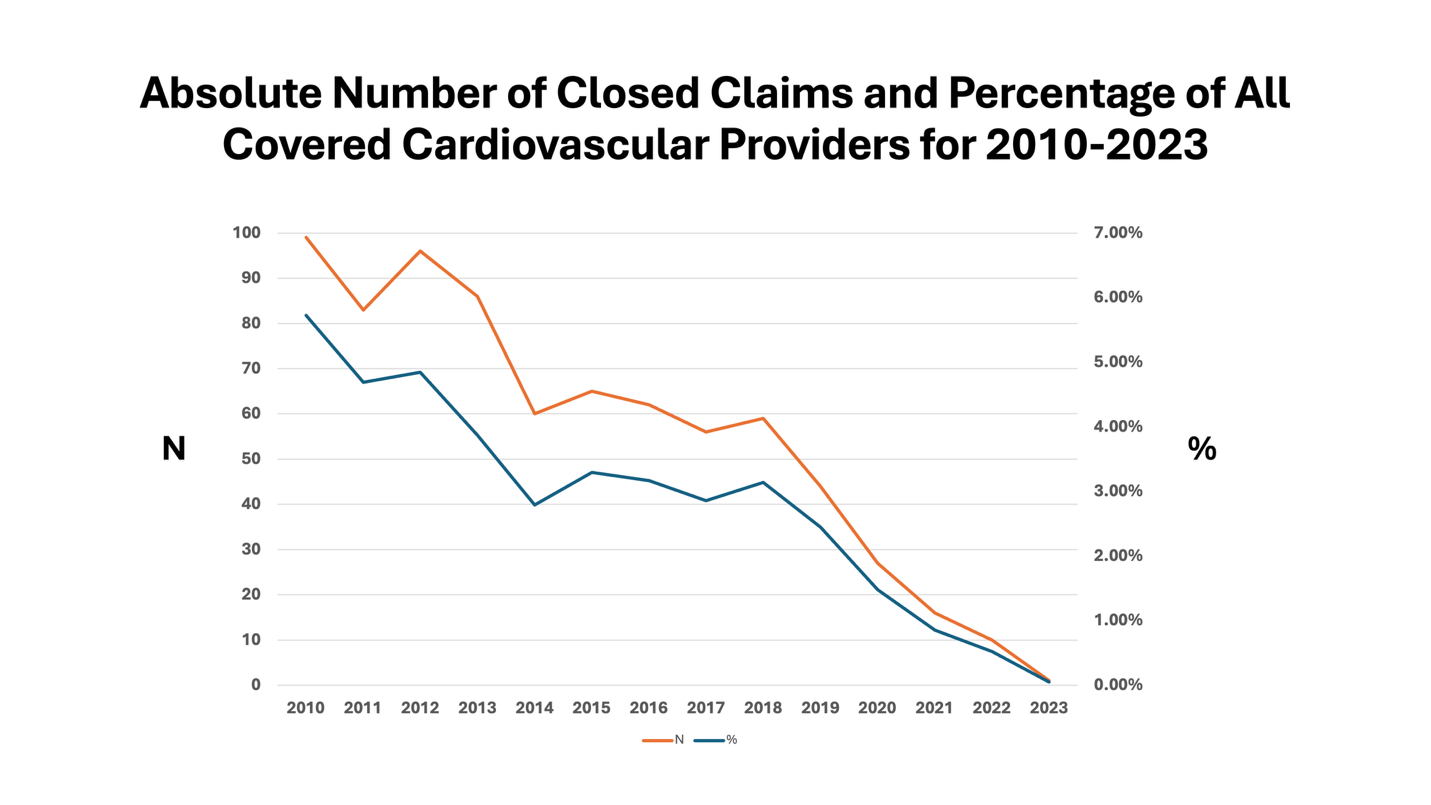


**Sup Figure 1:** Number of closed claims included in the study during the study period (2010-2023). The percentage of closed claims relative to the number of covered providers decreased from 4-5% to plateau at 3% between 2014-2019 (2020-2023 not included because of the COVID-19 pandemic and because closed claims are lagging indicators).


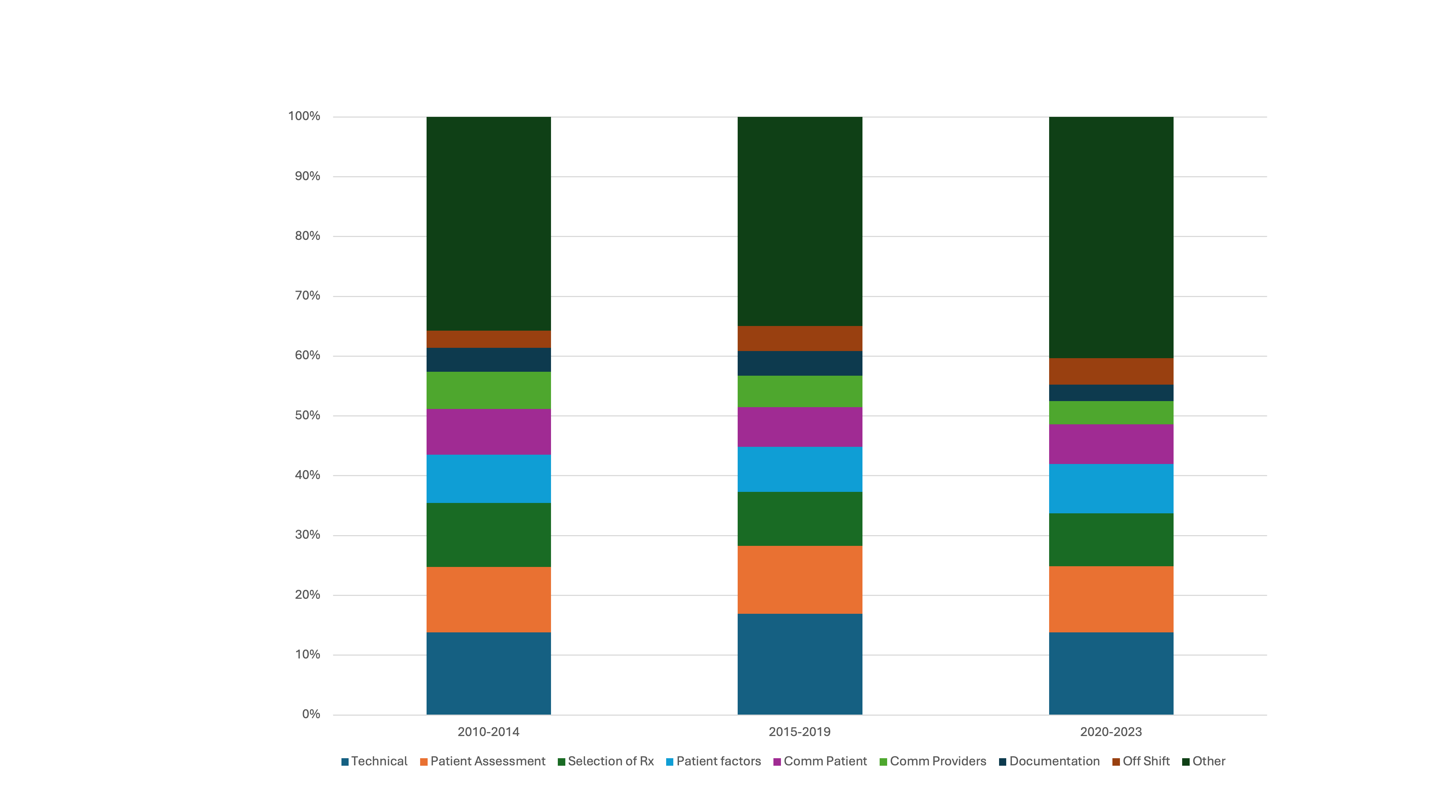


**Sup Figure 2:** No temporal changes for contributing factors among the three time strata were identified


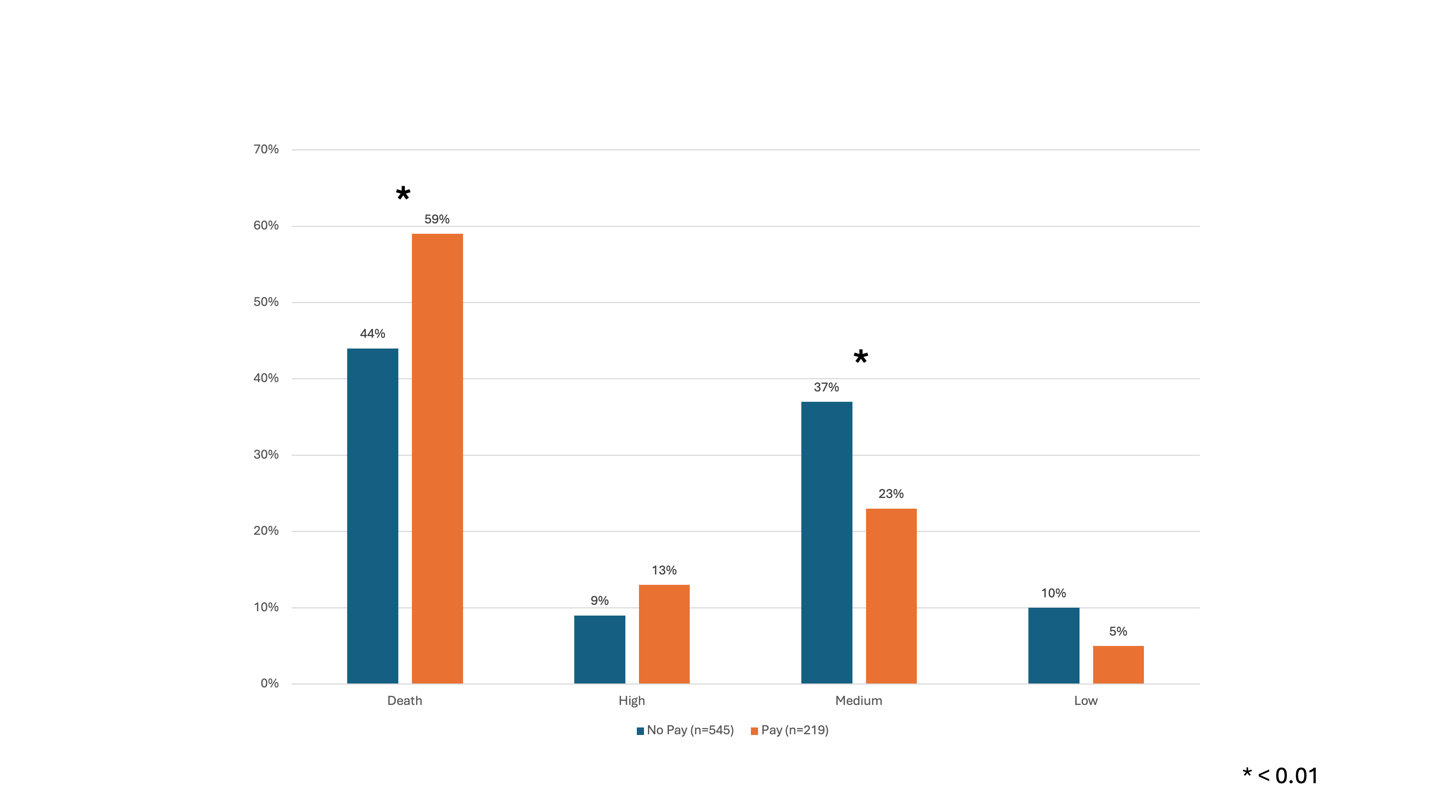


**Sup Figure 3:** Relationship between Injury severity and indemnity payment. Death was more likely associated with payment when compared to less severe levels of injury.


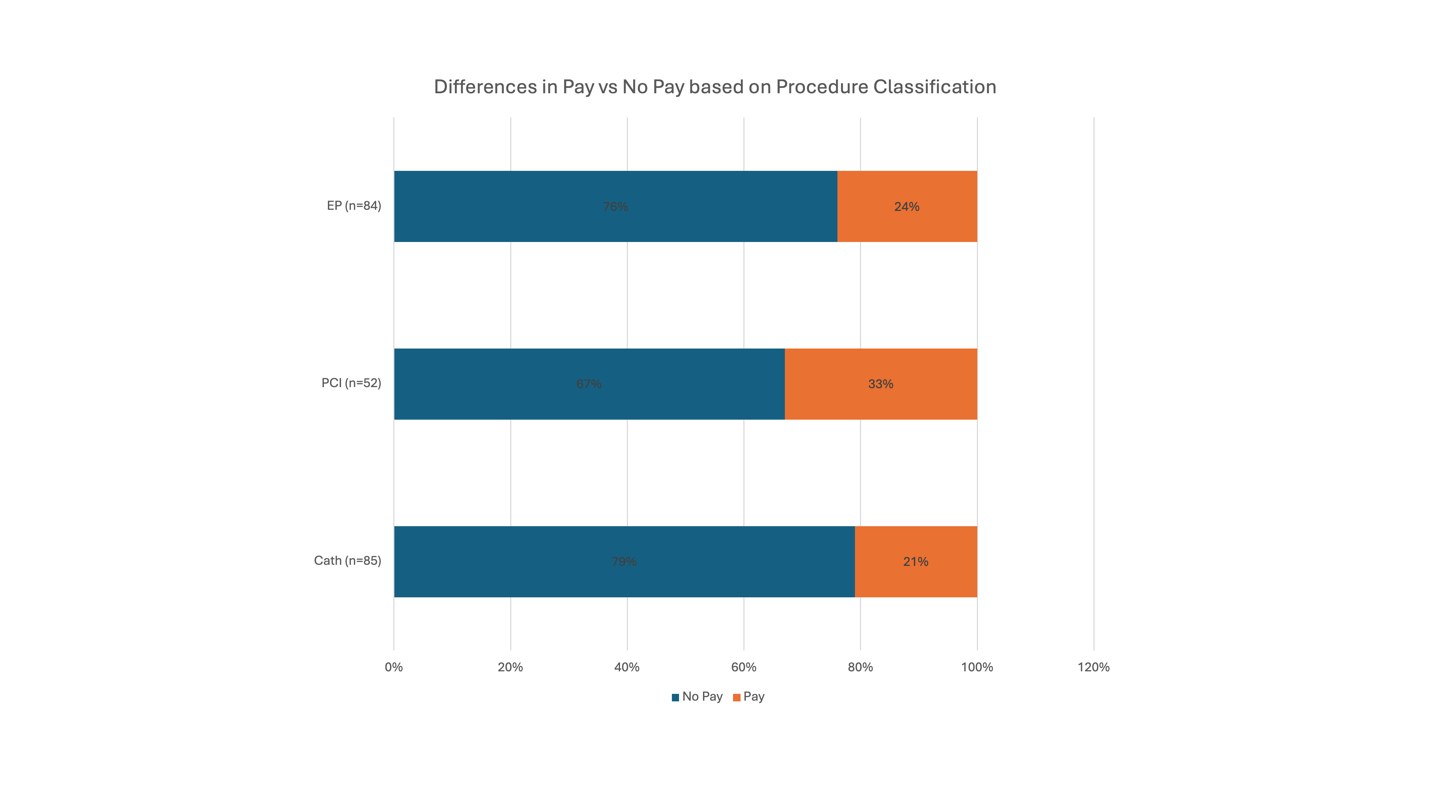


**Sup Fig 4:** No differences in frequency of indemnity payment were observed among different procedure types.


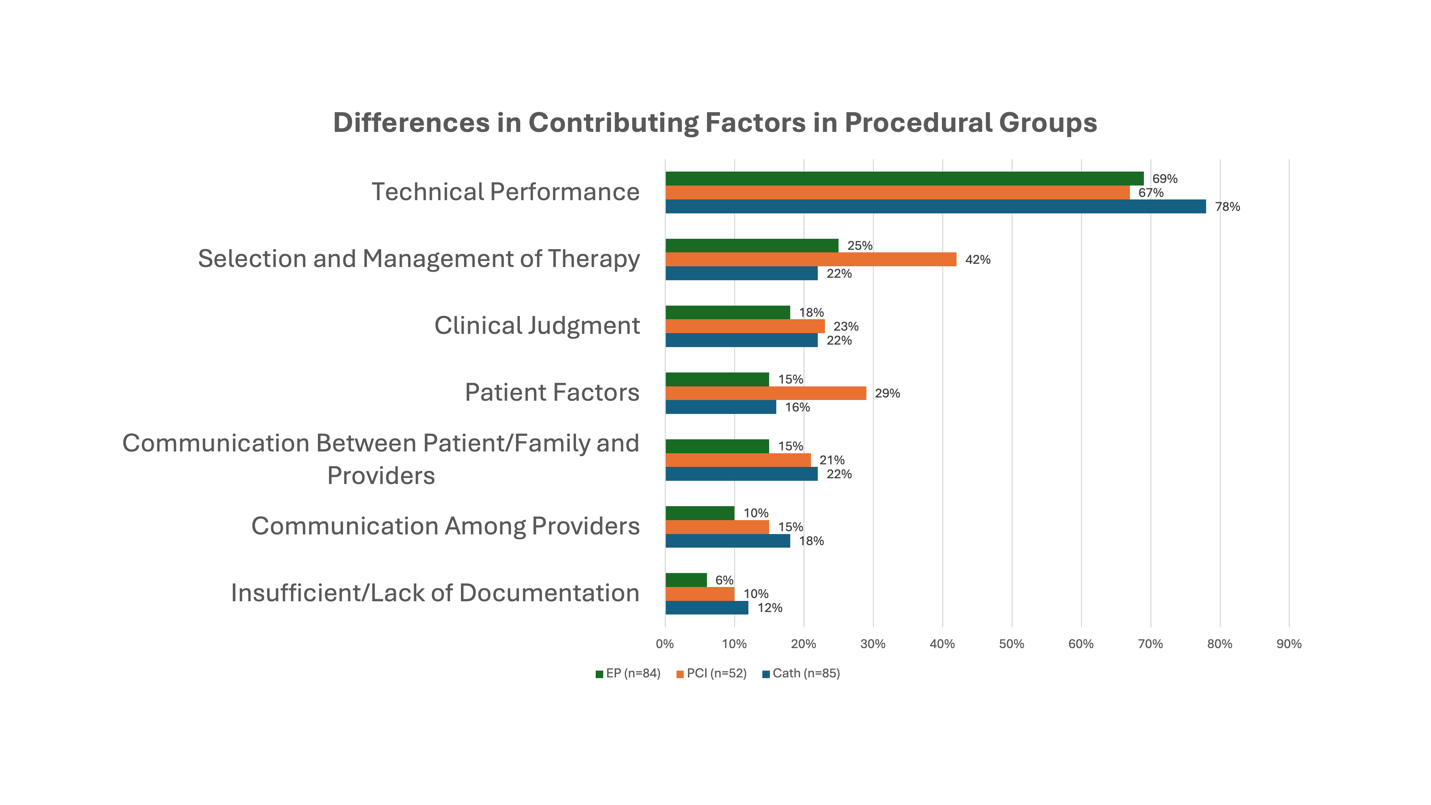


**Sup Fig 5:** No differences in contributing factors among specific cardiovascular procedures
